# No difference found in the magnetic resonance imaging signal intensity of the anterior cruciate ligament reconstruction graft between single‐bundle anterior cruciate ligament reconstruction with concomitant anterolateral ligament surgery and double‐bundle anterior cruciate ligament reconstruction

**DOI:** 10.1002/jeo2.70542

**Published:** 2025-11-27

**Authors:** W. P. Yau

**Affiliations:** ^1^ Department of Orthopaedics and Traumatology, School of Clinical Medicine, Li Ka Shing Faculty of Medicine The University of Hong Kong Hong Kong China

**Keywords:** DBACLR, MRI, SBACLR‐ALLR, SNQ

## Abstract

**Purpose:**

The purpose is to investigate whether the graft of single‐bundle anterior cruciate ligament reconstruction with concomitant anterolateral ligament surgery (SBACLR‐ALLR) has a healing advantage over that of double‐bundle ACLR (DBACLR). It is hypothesised that there is no difference in the signal‐to‐noise quotient (SNQ) of the ACLR graft between SBACLR‐ALLR and DBACLR.

**Methods:**

A retrospective case‐control study was conducted from 2010 to 2019, comparing patients who received SBACLR‐ALLR and DBACLR. The primary outcome was the SNQ of the ACLR graft. The secondary outcomes were the clinical results assessed at 2 years postoperation.

**Results:**

Forty‐eight SBACLR‐ALLRs and sixty DBACLRs with a mean follow‐up of 53 months were identified. The SNQ of the ACLR graft in SBACLR‐ALLR was comparable to that in DBACLR (6.9 ± 5.3 and 6.4 ± 5.0, respectively; *p* = 0.31). The graft rupture rates were 2.1% and 5%, respectively (*p* = 0.43). There was no statistically significant difference in the rate of return to previous sport at 2 years postoperation (SBACLR‐ALLR = 79%, DBACLR = 60%; *p* = 0.05). The 2‐year International Knee Documentation Committee (IKDC) subjective scores were 89.3 ± 9.2 and 87.6 ± 9.6, respectively (*p* = 0.21). Eighty‐four per cent of SBACLR‐ALLRs compared to 90% of DBACLRs achieved MCID in IKDC at 2‐year follow‐up (*p* = 0.33). There was no difference between the two groups in terms of the results of 2‐year Lachman, anterior drawer and pivot shift tests.

**Conclusion:**

When comparing SBACLR‐ALLR and DBACLR, no difference was found in the postoperative SNQ, the 2‐year graft rupture rate, the rate of return to sport, the proportion of patients achieving MCID in IKDC at 2‐year follow‐up, and the 2‐year clinical outcomes. These results suggest that adding an ALLR to SBACLR yields outcomes comparable to the more technically demanding DBACLR.

**Level of Evidence:**

Level III.

**Abbreviations**
ACL: anterior cruciate ligament
ACLR: anterior cruciate ligament reconstruction
ACLR‐ALLR: concomitant anterior cruciate ligament reconstruction and anterolateral ligament surgery
ALL: anterolateral ligament
AMB: anteromedial bundle
BMI: body mass index
DBACLR: double‐bundle anterior cruciate ligament reconstruction
DICOM: Digital Imaging and Communications in Medicine
EUA: examination under anaesthesia
ICC: intraclass correlation
IKDC: International Knee Documentation Committee
MCID: minimal clinically important difference
MRI: magnetic resonance imaging
PLB: posterolateral bundle
SBACLR: single‐bundle anterior cruciate ligament reconstruction
SBACLR‐ALLR: single‐bundle anterior cruciate ligament reconstruction with concomitant anterolateral ligament surgery
SNQ: signal‐to‐noise quotient
SPSS: Statistical Package for the Social Sciences
T: tesla
TAS: Tegner activity scale

## INTRODUCTION

Patients with anterior cruciate ligament (ACL) deficiency are treated with anterior cruciate ligament reconstruction (ACLR), either in the form of single‐bundle ACLR (SBACLR) [31], double‐bundle ACLR (DBACLR) [42], or concomitant ACLR and anterolateral ligament surgery (ACLR‐ALLR) [6, 8, 11, 20, 22, 30, 40]. The implanted graft in ACLR undergoes a series of histological and biochemical changes during the healing process, which is lengthy and may take more than 2 years to complete [2, 13]. The progress of healing can be assessed by observing the signal change of the ACLR graft in postoperative magnetic resonance imaging (MRI), the signal‐to‐noise quotient (SNQ) [35]. A high SNQ of the intact ACLR graft in the 2‐year postoperative MRI is associated with an increased likelihood of subsequent graft rupture [39].

SNQ is a measure of the signal intensity of the graft on the postoperative MRI. The amount of graft hyperintensity is related to the degree of graft oedema and the extent of re‐vascularisation that occur during healing of the ACLR graft [39]. The SNQ of the ACLR graft in the early post‐ACLR period is significantly higher than that of the native ACL [19]. It was shown in large animal model that there was a progressive reduction in SNQ from 6 to 52 weeks after ACLR. The decrease in SNQ parallels the histological changes of the ACLR graft during graft healing and remodelling [35]. After the remodelling of the ACLR graft is completed, the SNQ of the ACLR graft becomes comparable with that of the native ACL in both animal [35] and human studies [19]. Because of its association with graft healing and the noninvasive nature of the investigation, SNQ of the ACLR graft measured using postoperative MRI has been extensively used as a research tool to study the influence of co‐variates in the healing of the ACLR graft [26, 39].

Despite the results of SBACLR being generally good, residual laxity and repeated knee injury are not rare [31]. When compared to DBACLR, most randomised controlled trials found improved rotational control in the patients who receive DBACLR compared to SBACLR at short‐term follow‐up, while no differences were observed in the mid‐term to long‐term assessments [1, 15, 28, 32, 37]. When considering revision surgery, more technical concerns arise in failed DBACLR compared to failed SBACLR [10, 12].

To improve the rotational control of the knee, there has been increasing interest in reconstructing the anterolateral ligament (ALL) in patients with ACL deficiency [6, 8, 11, 20, 22, 30, 40]. The results of meta‐analysis showed SBACLR‐ALLR produces better clinical outcomes than isolated SBACLR, with a lower risk of graft rupture and fewer residual pivot shift phenomena [20, 21]. Cavaignac et al. reported that concomitant lateral extra‐articular tenodesis (LET) may accelerate graft maturation, as reflected by lower SNQ values [4], whereas Rojas et al. found that SNQ can be higher in 10‐month postoperative MRI when concomitant LET is performed [24]. The improved stability provided by the addition of ALL surgery in SBACLR‐ALLR could hypothetically promote better graft maturation [4]. However, it is also possible that the reconstructed ALL induces a stress shielding effect on the ACLR graft. The reduction in graft loading may negatively impact the remodelling process [24]. Currently, there is no consensus regarding the influence of anterolateral ligament surgery on the SNQ of the ACLR graft in ACLR.

Despite the abundance of publications comparing either SBACLR with DBACLR or SBACLR with SBACLR‐ALLR, there is limited literature comparing SBACLR‐ALLR with DBACLR [15, 16, 23]. Currently, there are no studies comparing the healing of the ACLR graft in terms of SNQ in the postoperative MRI between the two groups. It remains unknown whether a difference exists in the healing of the ACLR graft between SBACLR‐ALLR and DBACLR.

The purpose of this study is to investigate whether the graft of SBACLR‐ALLR has a healing advantage over that of DBACLR in terms of the SNQ of the intact ACLR graft in postoperative MRI. It is hypothesised that there is no difference in the SNQ of the ACLR graft between SBACLR‐ALLR and DBACLR.

## METHODS

A retrospective case‐control study comparing SBACLR‐ALLR and DBACLR with prospectively collected data was conducted at a single institution from July 2010 to January 2019. The study was approved by the local ethics committee (document number: UW 23‐163). The inclusion and exclusion criteria were summarised in Table 1. Ever‐smokers are excluded because it has been reported that smoking is associated with higher SNQ of the intact graft [5] and an increased risk of graft rupture [38].

**Table 1:** Inclusion and exclusion criteria.

Inclusion criteria
1. Patients underwent primary ACLRs between July 2010 and January 2019.
2. Patients were skeletally mature, as shown on preoperative knee radiographs.
3. Medial hamstrings autografts were used as the graft for ACLR.
4. Patients received a postoperative MRI.
5. Regarding the SBACLR‐ALLR cohort, concomitant ALLR was performed and the ACLR graft consisted of a quadrupled graft with two strands of the semitendinosus and two strands of the gracilis tendons.
6. Regarding the DBACLR cohort, concomitant ALLR was not performed and the configurations of the DBACLR were two strands of the semitendinosus tendons for the AMB graft and two strands of the gracilis tendons for the PLB graft.
EXCLUSION CRITERIA
1. Patients had generalised ligamentous laxity, as defined by a modified Beighton score of 5 or above.
2. Patients had multiple ligament injuries requiring concomitant ligament reconstruction, other than ACLR or ALLR.
3. Patients suffered from concomitant systemic inflammatory joint disease, including ankylosing spondylitis and rheumatoid arthritis.
4. Patients were ever‐smokers, including both active smokers and ex‐smokers.

Abbreviations: ACLR, anterior cruciate ligament reconstruction; ALLR, anterolateral ligament reconstruction; AMB, anteromedial bundle; DBACLR, double‐bundle anterior cruciate ligament reconstruction; PLB, posterolateral bundle; SBACLR‐ALLR, single‐bundle anterior cruciate ligament reconstruction with concomitant anterolateral ligament reconstruction; SNQ, signal‐noise quotient.

### Preoperative assessment

All the patients were assessed in a preoperative assessment clinic 1 week before the surgery. The following information was collected prospectively using a standard research documentation form: demographic data, Tegner activity scale (TAS), International Knee Documentation Committee (IKDC) subjective score, findings of physical examination and KT‐1000 measurements of anteroposterior laxity in both knees. ACL laxity, assessed through the Lachman test, anterior drawer test and pivot shift test, was graded according to the IKDC grading system: 0 (normal), 1+ (nearly normal), 2+ (abnormal) and 3+ (severely abnormal).

### Surgical procedure

The surgery was performed by two sports medicine surgeons, including the author (W.P.Y.). Ipsilateral semitendinosus and gracilis autografts were harvested. The tibial attachments of the medial hamstrings were not preserved.

### Surgical procedure—DBACLR

The anteromedial bundle (AMB) graft of DBACLR consisted of a two‐stranded semitendinosus tendon graft, and the posterolateral bundle (PLB) graft consisted of a two‐stranded gracilis tendon graft. The femoral tunnel was prepared using an anteromedial portal technique with the knee in maximum hyperflexion for both the AMB and PLB. The grafts were fixed with adjustable titanium buttons in the femur (Rigidloop, Depuy Synthes) and bioabsorbable interference screws in the tibia (Milargo Advance, Depuy Synthes). The fixation of the AMB graft was performed with the knee flexed at 30 degrees, while the fixation of the PLB graft was performed at 0‐degree extension.

### Surgical procedure—SBACLR‐ALLR

SBACLR‐ALLR has been performed since 2014. The ACLR graft used was a four‐stranded graft composed of two strands of the semitendinosus tendon and two strands of the gracilis tendon. Two surgical techniques were used for performing SBACLR‐ALLR:

2014–2016 Technique:

During this period, the femoral tunnel for SBACLR was prepared using an anteromedial portal technique with the knee in maximum hyperflexion. The SBACLR graft was fixed at 30‐degree knee flexion with one adjustable titanium button (Rigidloop, Depuy Synthes) and one bioabsorbable interference screw (Milargo Advance, Depuy Synthes). The ALLR graft was a two‐stranded graft prepared by doubling a free iliotibial band autograft. The iliotibial band was harvested via a longitudinal incision over the lateral aspect of the distal thigh. The central part of the iliotibial band was harvested as a free graft from just proximal to the lateral femoral epicondyle to the proximal thigh using a minimally invasive iliotibial band harvester. The typical dimensions of the harvested iliotibial band autograft were 10 mm wide and 200 mm long. The femoral footprint of the ALLR was prepared just proximal and posterior to the lateral femoral epicondyle, while the tibial footprint was at the Gerdy tubercle. The ALLR graft was fixed with suture anchors (Swivelock 4.75, Arthrex Inc) with the knee flexed at 30 degrees and in neutral rotation, taking care not to over‐tension the ALLR graft.

2017–2019 Technique:

During this period, the femoral tunnel for SBACLR was prepared using an outside‐in technique with the knee in 90‐degree flexion. A lateral longitudinal incision was made in the distal thigh to expose the lateral cortex of the distal femur and the femoral footprint of the ALLR. The ACLR graft was fixed with one bioabsorbable interference screw (Milargo Advance, Depuy Synthes) and one adjustable titanium button (Rigidloop, Depuy Synthes) with the knee flexed at 30 degrees. The ALLR graft was a one‐stranded graft, prepared using the remaining length of the semitendinosus tendon. The minimum lengths of the ACLR and ALLR grafts were 70 and 100 mm, respectively. The femoral footprint of the ALLR was located just proximal and posterior to the lateral femoral epicondyle, while the tibial footprint was at the Gerdy tubercle. The ALLR graft was fixed with suture anchors (Biocomposite Corkscrew suture anchor, Arthrex Inc) with the knee flexed at 30 degrees and in neutral rotation, taking care to avoid over‐tensioning the ALLR graft.

### Follow‐up and clinical assessment

Patients were followed up at a designated ACL reconstruction clinic every 3 months in the first year, and then annually. Clinical data, including TAS, IKDC subjective score and physical examination findings, were prospectively collected during the follow‐up. The data were collected by an independent observer, who was not the operating surgeon. However, neither the independent observer nor the patient was blinded to the surgical technique. Patients were considered to have returned to their previous level of sport if their 2‐year TAS was the same or higher than their preinjury TAS. Graft rupture was diagnosed either through postoperative MRI or repeated arthroscopy.

### Postoperative MRI

All patients underwent postoperative MRI, typically scheduled for the second year after surgery. The MRI was performed using a 1.5 T machine with a slice thickness of 3 mm and a matrix of 512 × 512 at the author's institute. The images were acquired in sagittal, coronal and axial planes. 3D MRI was not utilised. It has been reported that the SNQ of an intact ACLR graft, measured using MRI performed in the second year after surgery, is most predictive of future graft rupture [39]. The rationale for performing MRI in the second year is that the primary aim of routine postoperative imaging for all patients undergoing ACLR at the authors' institution is to identify individuals at risk of future graft rupture.

### SNQ measurement

**Figure 1:**
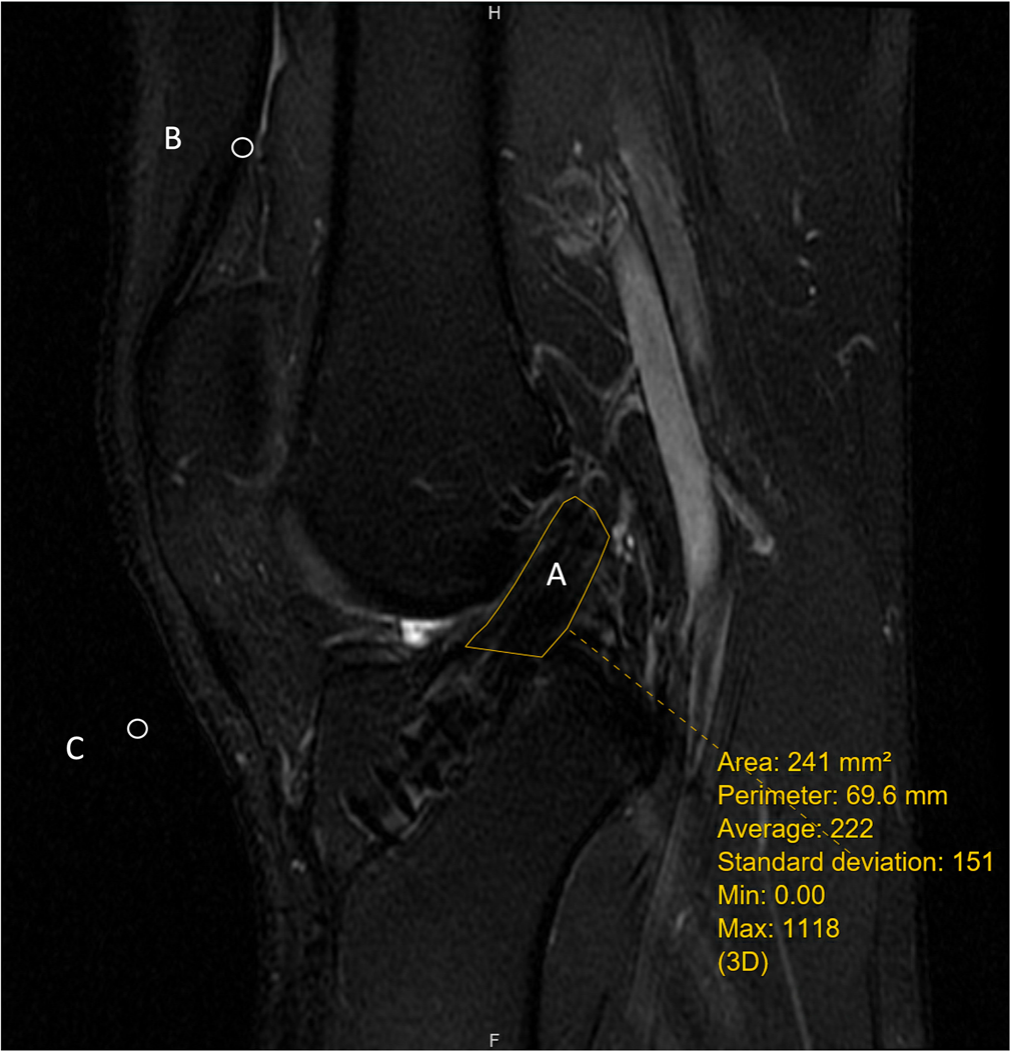
Signal‐to‐noise quotient of the intra‐articular portion of the anterior cruciate ligament reconstruction graft. A: Signal of the graft of the anterior cruciate ligament reconstruction. B: Signal of the quadriceps tendon (taken at the quadriceps tendon 2 cm above the upper pole of patella). C: Signal of background (taken at the background 1 cm anterior to the patellar tendon). Signal‐to‐noise quotient = $\frac{\mathrm{Overall}\;\mathrm{signal}\;\mathrm{of}\;\mathrm{the}\;\mathrm{graft}-\mathrm{Signal}\;\mathrm{of}\;\mathrm{the}\;\mathrm{quadriceps}\;\mathrm{tendon}}{\mathrm{Signal}\;\mathrm{of}\;\mathrm{background}}.$

The degree of graft maturation in ACLR was measured if the graft was found to be intact in the postoperative MRI. The digital images of the MRI were input into the eUnity DICOM workstation and viewer (Mach7 Technologies Canada Inc.). The SNQ of the intra‐articular portion of the entire graft was measured in the T2 sagittal slice that provided the best visualisation of the ACLR graft [5] (Figure 1). It was normalised with reference to the signal of the quadriceps tendon and the noise of the background according to the equation SNQ = (overall signal of the graft – signal of the quadriceps tendon)/signal of the background. The outline of the intra‐articular portion of the ACLR graft was traced and the overall signal of the ACLR graft was measured. The signal of the quadriceps tendon was measured at a site 2 cm above the upper pole of the patella. The signal of the background was measured at a site 1 cm anterior to the patellar tendon. For DBACLR, only the signal of the AMB graft was measured. The orientations of the AMB and PLB are different—where the AMB runs more along the anterior‐posterior (AP) direction, while the PLB runs more along the medial‐lateral dimension. The AMB is well visualised in sagittal plane MRI images, making SNQ measurement easier and more reproducible. Conversely, the PLB is often less clearly seen in sagittal images, making SNQ measurement for it more challenging. As a result, only the SNQ of the AMB was measured.

### Inter‐ and intraobserver repeatability in measurement of SNQ

Interobserver repeatability in measuring the SNQ was assessed by having two independent observers (W.P.Y. and W.L.) measure the SNQ. Intraobserver repeatability was evaluated by taking repeated measurements of the SNQ 1 week after the first measurement. The repeatability was evaluated using the intraclass correlation (ICC). Repeatability was considered poor for an ICC < 0.4, fair for an ICC 0.4–0.59, good for an ICC 0.6–0.74 and excellent for an ICC 0.75–1.

### Clinical outcomes

The TAS, IKDC subjective score and presence of residual ACL laxity signs were reported for patients who attended the 2‐year follow‐up.

### Statistical analysis

The SNQ of the graft, the incidence of graft rupture within the first 2 years after surgery, and the 2‐year clinical outcomes were reported. The data were analysed using SPSS software (version 28) (IBM, Armonk). The student *t*‐test was used to analyse continuous data, while the chi‐square test was used for categorical data. Statistical significance was assumed if the *p*‐value was less than 0.05. Minimal clinically important difference (MCID) of IKDC was calculated using the 0.5‐SD method. The percentage of patients achieving MCID in IKDC at the 2‐year follow‐up was reported.

## RESULTS

**Figure 2:**
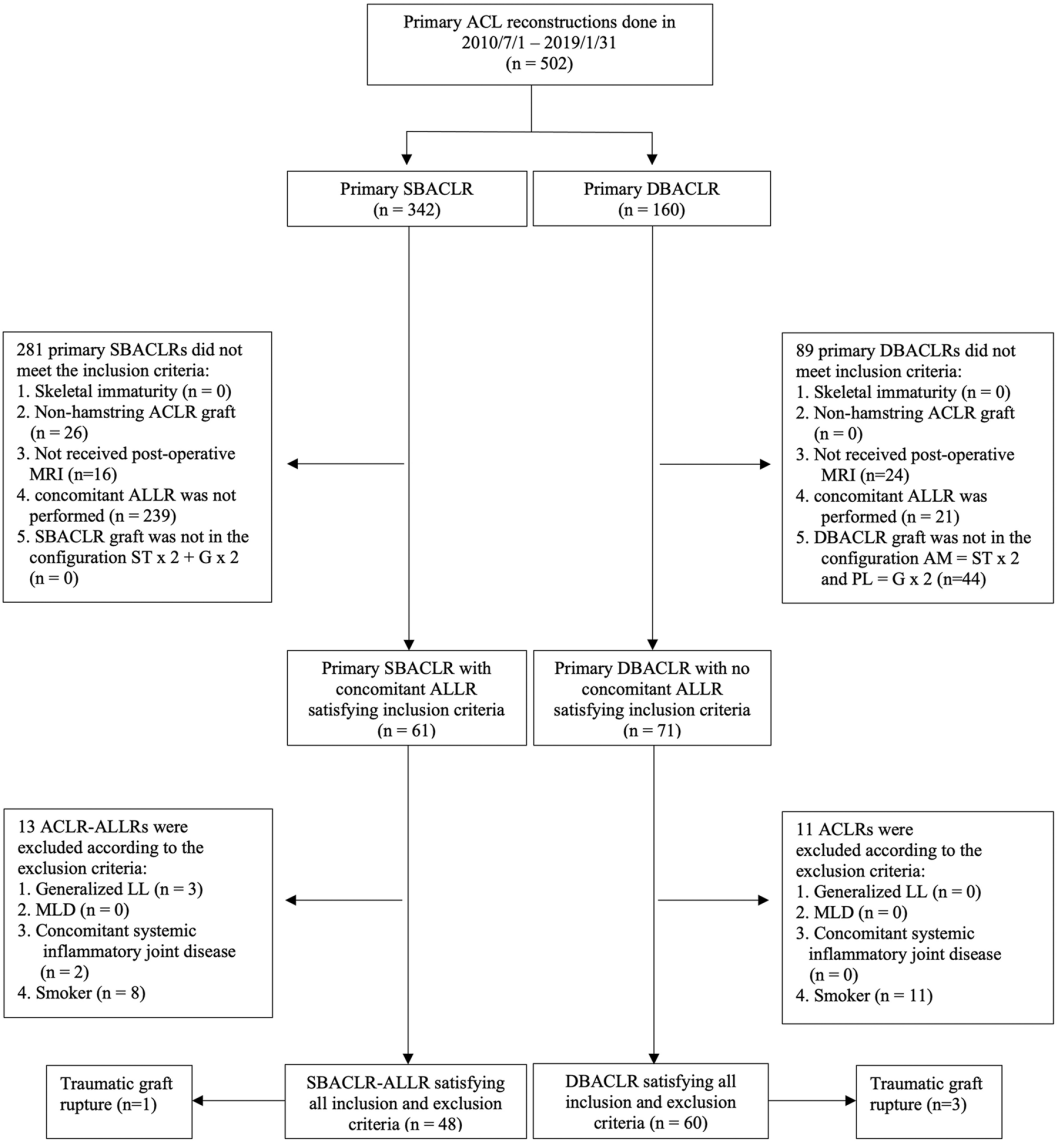
Enrollment of Subjects. ACLR, anterior cruciate ligament reconstruction; ALLR, anterolateral ligament reconstruction; AM, anteromedial bundle; DBACLR, double‐bundle anterior cruciate ligament reconstruction; G, gracilis; Generalised LL, generalised ligamentous laxity; MLD, multiple ligament deficiency with concomitant ligament reconstruction, other than anterolateral ligament reconstruction; N, number; PL, posterolateral bundle; SBACLR, single‐bundle anterior cruciate ligament reconstruction; SBACLR‐ALLR, single‐bundle anterior cruciate ligament reconstruction with concomitant anterolateral ligament reconstruction; ST, semitendinosus.

A total of 502 primary ACLRs were performed at the author's institute between July 2010 and January 2019 (Figure 2). Among these, 132 patients satisfied the inclusion criteria. One hundred and eight primary ACLRs satisfied all inclusion and exclusion criteria, including 48 SBACLR‐ALLRs and 60 DBACLRs. These included unpublished data from a randomised controlled trial comparing SBACLR‐ALLR and DBACLR, accounting for 28 SBACLR‐ALLRs and 23 DBACLRs. The allocation of surgical options in this group was determined by simple randomisation. The remaining 57 cases, including 20 SBACLR‐ALLRs and 37 DBACLRs, were retrospectively identified from a retrospective cohort. The allocation of surgery in this group was based on surgeon preference. The rate of two‐year follow‐up was 85% in the SBACLR‐ALLR group and 92% in the DBACLR group. There were four graft ruptures at or before the follow‐up of 2 years, including one rupture in the SBACLR‐ALLR and three ruptures in the DBACLR group (*p* = 0.43) (Figure 2).

The mean follow‐up was 53 months. The average follow‐up was longer in the DBACLR group compared to the SBACLR‐ALLR group (*p* < 0.01). Other than this, there was no difference between the two groups in terms of demographic data, time between injury and operation, preoperative ACL laxity and the need for concomitant meniscus surgery (Table 2).

**Table 2:** Demographic data and length of follow‐up.

	SBACLR‐ALLR	DBACLR	*p* value
Number	48	60	‐
Follow‐up (months)	39 ± 22	64 ± 39	*p* < 0.001*
Time between MRI and surgery (months)	19 ± 10	20 ± 9	*p* = 0.21
Age	25.3 ± 5.9	25.8 ± 6.7	*p* = 0.35
Sex (male vs. female)	48 versus 0	57 versus 3	*p* = 0.16
Laterality (right vs. left)	24 versus 24	35 versus 25	*p* = 0.39
BMI (kg/m^2^)	24.5 ± 3.1	24.5 ± 3.4	*p* = 0.48
Preinjury Tegner activity scale	6.6 ± 1.2	6.6 ± 1.4	*p* = 0.44
Time between injury and surgery (days)	376 ± 750	607 ± 1246	*p* = 0.13
EUA Pivot shift 3	2 out of 48	2 out of 60	*p* = 0.82
Preoperative KT‐1000 side‐to‐side difference at 30 lb (mm)	3.0 ± 2.0	3.6 ± 2.2	*p* = 0.11
Medial meniscus: meniscectomy versus repair versus no tear	7 versus 16 versus 25	9 versus 19 versus 32	*p* = 0.98
Lateral meniscus: meniscectomy versus repair versus no tear	6 versus 12 versus 30	10 versus 18 versus 32	*p* = 0.62

Abbreviations: BMI, body mass index; DBACLR, double‐bundle anterior cruciate ligament reconstruction; EUA, examination under anaesthesia; MRI, magnetic resonance imaging; SBACLR‐ALLR, single‐bundle anterior cruciate ligament reconstruction with concomitant anterolateral ligament surgery.

* Statistical significant.

### SNQ

The mean SNQs of the ACLR graft of SBACLR‐ALLR and the AMB of DBACLR were 6.9 ± 5.3 and 6.4 ± 5.0, respectively. There was no difference between the two groups (*p* = 0.31). The 95% confidence interval (CI) for the difference was from −1.5 to 2.5.

The inter‐ and intraobserver repeatability in the measurement of SNQ were both excellent, with an interobserver ICC of 0.8 and an intraobserver ICC of 0.96.

### Clinical results

There was no statistically significant difference in the rate of return to premorbid sport between the SBACLR‐ALLR group and the DBACLR group (79% and 60%, respectively; *p* = 0.05). There was no difference between the two treatment groups in terms of the 2‐year TAS, the mean IKDC subjective score, and the presence of grade 2/3 residual Lachman, anterior drawer test and pivot shift test at the 2‐year follow‐up (Table 3). The MCID of IKDC was 7.5. Eighty‐four per cent of patients with SBACLR‐ALLR achieved MCID in IKDC at the 2‐year follow‐up, compared to 90% of patients with DBACLR (*p* = 0.33).

**Table 3:** Clinical outcomes at 2‐year follow‐up.

	SBACLR‐ALLR	DBACLR	*p* value
Number of patients with 2‐year follow‐up (excluding graft rupture)	40	52	‐
Tegner activity scale at 2‐year follow‐up	5.9 ± 1.9	5.4 ± 2.1	*p* = 0.10
Percentage of patient return to previous sport level at 2‐year follow‐up	79%	60%	*p* = 0.05
IKDC at 2‐year follow‐up	89.3 ± 9.2	87.6 ± 9.6	*p* = 0.21
Proportion of patients who achieve MCID in IKDC at the 2‐year follow‐up	84%	90%	*p* = 0.33
Lachman test at 2‐year follow‐up (0/1+ vs. 2+/3+)	95% versus 5%	88% versus 12%	*p* = 0.24
Anterior drawer test at 2‐year follow‐up (0/1+ vs. 2+/3+)	90% versus 10%	83% versus 17%	*p* = 0.25
Pivot shift test at 2‐year follow‐up (0/1+ vs. 2+/3+)	93% versus 7%	92% versus 8%	*p* = 0.65

*Note*: ‐, statistical test not performed. Abbreviations: ACLR, anterior cruciate ligament reconstruction; DB‐ACLR, double bundle anterior cruciate ligament reconstruction; IKDC, International Knee Documentation Committee Subjective Score; SBACLR‐ALLR, single bundle anterior cruciate ligament reconstruction with concomitant anterolateral ligament surgery.

## DISCUSSION

There was no difference between the SNQ of the ACLR graft of SBACLR‐ALLR and that of the AMB graft of DBACLR. Additionally, the rate of graft rupture, the incidence of return to preinjury sport at the 2‐year follow‐up, and the 2‐year clinical outcomes were comparable between the two groups. However, these findings should be interpreted with caution due to the relatively small sample size of this study.

SNQ of the ACLR graft measured using postoperative MRI has been extensively used as a research tool to study the influence of co‐variates in the healing of the ACLR graft [4, 14, 24, 33, 39]. The SNQ of the ACLR graft in the early post‐ACLR period is significantly higher than that of the native ACL [19, 35, 39]. After the remodelling of the ACLR graft is completed, the SNQ of the ACLR graft becomes comparable with that of the native ACL in both animal [35] and human studies [19]. The expected time for the SNQ of the ACLR graft to return to a level comparable to that of the native ACL is around 2–4 years [19, 39]. The result of the current study shows that the augmentation of SBACLR with concomitant ALLR does not improve the SNQ of the ACLR graft compared to the AMB graft of DBACLR, showing that there is no difference in the healing of the ACLR graft between SBACLR‐ALLR and DBACLR.

There is controversy in the literature about the influence of anterolateral augmentation on the postoperative SNQ of the ACLR graft [4, 24, 41]. Cavaignac et al. reported that the average SNQ of the ACLR graft was smaller in the concomitant SBACLR‐ALLR study group compared to the isolated SBACLR control group [4]. Ye et al. reported that SNQ was significantly lower in the AMB of DBACLR in the group with combined DBACLR and anterolateral surgery, compared to that of isolated DBACLR [41]. On the other hand, Rojas et al. found that concomitant anterolateral augmentation resulted in a poorer SNQ of the ACLR graft in postoperative MRI [24]. Currently, there is no consensus on the influence of ALLR on the SNQ of the ACLR graft.

The rate of return to premorbid sport at the 2‐year follow‐up in the current study was 79% for SBACLR‐ALLR and 60% for DBACLR (*p* = 0.05). In this study, the rate of return to preinjury sport in DBACLR was comparable to the pooled value reported in the systematic review of the literature [36]. Regarding SBACLR‐ALLR, Sonnery‐Cotte et al. reported higher odds of returning to the preinjury level of sport in SBACLR‐ALLR compared to SBACLR (68.8% and 59.9%, respectively; odds ratio, 1.9; 95% CI, 1.2–3.2) [30]. The rate of return to preinjury sport of 79% in the SBACLR‐ALLR group of the current study compared favourably to the reported data in the Sonnery‐Cotte et al.'s publication. The borderline *p*‐value of 0.05 observed in this study suggests that the failure to achieve statistical significance in the difference in return‐to‐sport rates between the SBACLR‐ALLR and DBACLR groups is likely due to the small sample size. Reported rates of return to preinjury sport in the literature vary widely, ranging from 33% to 100%, with a pooled rate of 63% [3]. Multiple factors influence a patient's ability to return to sport after ACLR, including knee stability, muscle strength, proprioception recovery [3] and psychological readiness to resume activity [3, 36]. Fear of re‐injury has been identified as the most common reason for postoperative reduction or cessation of sports participation [3]. Currently, there is no consensus in the literature regarding the most effective postoperative rehabilitation protocol to facilitate return to sport—whether accelerated, standardised, or criterion‐based [18]. In this study, no significant differences were found between the SBACLR‐ALLR and DBACLR groups concerning patient characteristics, graft rupture rates within 2 years postoperation, selfreported symptoms, or physical signs of ACL deficiency observed during physical examinations at the 2‐year follow‐up. Although the study spanned nearly 10 years, the same standardised rehabilitation protocol was applied to all patients. Because psychological assessments were not conducted, it remains unknown whether differences in psychological readiness exist between the two groups.

The incidence of a grade 0/1 pivot shift test at the 2‐year follow‐up was 93% for SBACLR‐ALLR and 92% for DBACLR in the current study (*p* = 0.65). There was no difference in the 2‐year IKDC subjective score between the two groups (89.3 ± 9.2 and 87.6 ± 9.6, respectively, *p* = 0.21). Eighty‐four per cent of patients with SBACLR‐ALLR achieved MCID in IKDC, compared to 90% of patients with DBACLR (*p* = 0.33). These findings are similar to the conclusions of available literature comparing SBACLR‐ALLR and DBACLR, indicating no significant difference in the clinical outcomes between the two groups [15, 16, 23].

Graft rupture is a possibility after both SBACLR‐ALLR and DBACLR [20, 21, 28], and the incidence of graft rupture after ACLR is expected to increase with extended follow‐up times [9, 17, 25, 27]. Other than the rare scenario of infection, whether the revision can be performed as a one‐ or two‐stage procedure depends on the position of the original bone tunnels and the presence of significant tunnel widening at the time of revision surgery [7]. There is concern about the technical difficulty in revising a failed DBACLR because two bone tunnels have been created in both the femur and tibia, which potentially increases the risk of tunnel malposition and tunnel widening [10, 12]. Tunnel communication between the AMB and PLB bone tunnels presents a specific surgical consideration in the setting of failed DBACLR, occurring at rates of 50% and 23% in the distal femur and proximal tibia, respectively [34]. Nevertheless, meticulous prerevision surgical planning with a CT scan of the knee has helped reduce the incidence of two‐stage revisions to 5% of failed DBACLR cases [29]. Considering the comparable clinical results between SBACLR‐ALLR and DBACLR, as well as the greater technical difficulty in revising a failed DBACLR [10, 12], SBACLR‐ALLR may be regarded as a more favourable option than DBACLR, especially for young, active patients who are known to be at risk of graft rupture and revision surgery [30].

## LIMITATIONS

First, postoperative MRI was not conducted at the exact same time point for all patients, which contributed to bias in the measurement of the SNQ. SNQ was measured only for the AMB graft in the DBACLR, and not for the PLB graft. There may be differences in SNQ between these two bundles, so the SNQ value obtained for the DBACLR may not be as representative as that for the SBCLR‐ALLR group, which could introduce bias into the statistical analysis. Furthermore, the postoperative MRIs were performed in the second year after surgery. However, most studies investigating ligamentization have conducted SNQ measurements during the 6‐month to 1‐year period, when remodelling activity is most intense and SNQ differences are more prominent [4, 24]. Readers should be aware of the potential bias introduced by the difference in timing of postoperative MRI in our study compared to the literature. Second, interobserver and intraobserver errors for the physical examination result were not available, and the extent of bias in these measurements remained unknown. In addition, neither the independent observer nor the patient was blinded to the surgical technique of the ACLR procedures. Third, nearly all the included patients were men. Therefore, it is not appropriate to extrapolate the findings of this study to other cohorts consisting mainly of women. Forth, smokers have been excluded. Thus, the generalisability of the results of this study may be limited for populations with a high prevalence of smoking. Fifth, the method of allocation of SBACLR‐ALLR or DBACLR in this retrospective study was not standardised. The decision regarding surgery was based on surgeon preference in the 57 cases identified from the retrospective cohort, while the remaining 51 cases, derived from the unpublished data of a randomised controlled trial comparing SBACLR‐ALLR and DBACLR, were assigned through simple randomisation. Selection bias was present. Additionally, the two groups are not comparable regarding the length of follow‐up. Sixth, the results indicating no statistically significant difference between the SBACLR‐ALLR and DBACLR groups regarding graft rupture rate, return to sport and clinical outcomes at 2 years postoperation should be interpreted with caution. Graft rupture occurred in one patient in the SBACLR‐ALLR group and in three patients in the DBACLR group. Although this difference is not statistically significant, it is notable in absolute numbers. The possibility that this study is underpowered cannot be ruled out. Additionally, 79% of patients who received SBACLR‐ALLR, compared to 60% of those who received DBACLR, were able to return to preinjury sport at the 2‐year follow‐up, with a borderline *p*‐value of 0.05. It is possible that statistical significance could be achieved with a larger sample size. Readers should be aware of the potential for a Type II error, despite these findings being consistent with the conclusions of other studies comparing SBACLR‐ALLR and DBACLR [15, 16, 23]. Seventh, the average follow‐up period for patients who received DBACLR was longer than that for those who underwent SBACLR‐ALLR, despite 2‐year follow‐up rates of 85% in the SBACLR‐ALLR group and 92% in the DBACLR group. This discrepancy may have introduced bias in the imaging and clinical outcomes.

## CONCLUSION

When comparing SBACLR‐ALLR and DBACLR, no difference was found in the postoperative SNQ, the 2‐year graft rupture rate, the rate of return to sport, the proportion of patients achieving MCID in IKDC at 2‐year follow‐up, and the 2‐year clinical outcomes. These results suggest that adding an ALLR to SBACLR yields outcomes comparable to the more technically demanding DBACLR.

## AUTHOR CONTRIBUTIONS

All authors contributed to the study conception and design. Material preparation, data collection and analysis were performed by W. P. Yau. The first draft of the manuscript was written by W. P. Yau and all authors commented on previous versions of the manuscript. All authors read and approved the final manuscript.

## CONFLICT OF INTEREST STATEMENT

The author declares no conflicts of interest.

## ETHICS STATEMENT

The current study involves human participants. The local ethics committee (Institutional Review Board of the University of Hong Kong/Hospital Authority Hong Kong West Cluster) approved and monitored the current study (Document number: UW 23‐163). The local ethics committee (Institutional Review Board of the University of Hong Kong/Hospital Authority Hong Kong West Cluster) waived the requirement for obtaining informed consent from the participants (Document number: UW 23‐163).

## Data Availability

The data of this study are available from author upon reasonable request and with permission of the local ethics committee.
